# Medicine in 1896

**Published:** 1897-02

**Authors:** 


### EDITORIAL.

The office of the Business Manager of THE JOURNAL is Nos. 311 and 312 Fitten Building.

The editors are not responsible for views expressed by contributors.

Articles for publication should reach this office not later than the 15th inst.

Address all communications, and make all remittances payable to THE ATLANTA MEDICAL AND SURGICAL JOURNAL, Atlanta, Ga.

Reprints of articles will be furnished, when desired, at a nominal cost. Requests for the same should always be made *on the manuscript.*

If a subscriber notifies us to stop his JOURNAL and it is not done, or, if he orders his address changed and it is not, he may know that we did not get his order. Moving to another address without notifying us, does not excuse him from paying his bill.

### MEDICINE IN 1896.

The Boston *Medical and Surgical Journal* is accustomed to present at the end of each year a concise review of the progress of medicine and surgery during the year. The review for 1896 is particularly interesting and justifies a condensed report of it here.

The year marked the centennial anniversary of Jenner's first successful vaccination, and in a number of places in all countries appropriate celebrations were held in honor of this important discovery. The semi-centennial (October 16) jubilee of anesthesia was celebrated in Boston in honor of the demonstration of anesthesia in the Massachusetts General Hospital fifty years previously.

Considerable use has been made of the Roentgen rays in medicine and surgery. The first application was the detection of foreign bodies in the tissues, particularly the extremities. Then with improved apparatus the deeper regions were explored, and bodies have been located in the esophagus, the gastro-intestinal canal, etc. Uric acid calculi were found to obstruct the rays, but gall-stones were transparent to them. But it is in the diagnosis and treatment of fractures and dislocations that the rays have found, and will probably continue to find, their most important application. One may now tell almost with the certainty that comes from direct observation whether a fracture or dislocation is properly reduced. The diagnosis of tumors of bones and of joint lesions has been greatly facilitated, affording most valuable aids to prognosis and treatment. The fluoroscope has thrown its light upon deep-seated chest affections, and tuberculosis of the lungs, pleurisy with effusion, aneurism of the aorta, dilatation of the heart, etc., have been made out with its help.

During the year smallpox has prevailed in certain sections of the country, but has usually been well controlled. Along the Mississippi and Ohio rivers it has existed, chiefly among the negro deck-hands on river steamers. In January and February it appeared in New Orleans and Memphis and began to spread rapidly. Steamer crews were vaccinated by sanitary inspectors, and by the spring there was a marked decline in the number of cases all along the Mississippi valley. A severe outbreak occurred among the negro population of Key West during June and July. A rigid quarantine was established and house-to-house vaccination was practiced by State and Marine Hospital officials and the epidemic was under control by the middle of August. Of course conditions incident to the war in Cuba have favored the existence and spread of smallpox. The crowding together of troops, the massing of the country population in large towns and the destitution caused by the war have favored the prevalence of both smallpox and yellow fever. The mortality in the Spanish military hospitals has been high. In Santiago alone there were thirty or forty deaths per week in July and August. In one week in Havana there were sixty-seven deaths. There were small outbreaks during the year in Arizona, Texas, Illinois, Kentucky and West Virginia. To some extent also it has existed in London, Gloucester, Constantinople and Rio de Janeiro.

Yellow fever has prevailed in the seaports of the West Indies, its continuance being favored by the disturbed conditions in the islands. Its ravages have been greatest among the Spanish soldiers. Smallpox has been more fatal among the native population. Our efficient quarantine regulations have preserved our own seaports against infection. Only one case of yellow fever landed on our shores during the year. This patient arrived at New York in October and was detained at the quarantine island, where he died in three days.

Antitoxin for diphtheria has continued to justify the hopes that have been placed in it. From the report of the American Pediatric Society's collective investigation we learned of 3,384 cases treated by 615 physicians, and of 2,400 cases treated by the Board of Health in New York and Chicago, and the mortality of the whole was only 12.3 per cent. It was clearly shown that the earlier the remedy was employed, the better the results and the lower the mortality. These statistics embrace the reports of a large number of physicians in widely separated localities, and they represent every variation as to local conditions, severity of the epidemics, etc., and they are more valuable therefore than statistics gathered from single institutions.

"From all quarters and under all conditions where the antitoxin treatment has been carried out, the reports issued during the year 1896 have fully sustained the claims made for it. There can be no doubt that thousands of lives have already been saved by this remedy, which may be justly termed the greatest contribution to the healing art made by modern scientific medicine."

